# Photodegradation of Dacarbazine Catalyzed by Vitamin B_2_ and Flavin Adenine Dinucleotide Under Visible-Light Irradiation

**DOI:** 10.1007/s11095-024-03802-2

**Published:** 2024-12-18

**Authors:** Yuka Kimura, Mayuko Suga, Kayo Nakamura, Hidetsugu Tabata, Tetsuta Oshitari, Hideaki Natsugari, Hideyo Takahashi

**Affiliations:** 1 Department of Medicinal Chemistry, Faculty of Pharmaceutical Sciences, Tokyo University of Science, 2641Yamazaki, Noda-Shi, Chiba, 278-8510 Japan; 2 Department of Medicinal Chemistry, Faculty of Pharma Sciences, Teikyo University, Itabashi-Ku, Tokyo, Japan; 3 Department of Medicinal Chemistry, Graduate School of Pharmaceutical Science, The University of Tokyo, Bunkyo-Ku, Tokyo, Japan

**Keywords:** dacarbazine, flavin adenine dinucleotide, photodegradation, riboflavin, vitamin B_2_

## Abstract

**Purpose:**

Drug photodegradation is a matter of great concern because it can result in potency loss and adverse side effects. This study examines the light-induced degradation of dacarbazine catalyzed by vitamin B_2_ and flavin adenine dinucleotide (FAD) under light-emitting diode (LED) or fluorescent light irradiation.

**Methods:**

Dacarbazine was irradiated with LED (405 nm) or fluorescent light in the presence of various equivalents of vitamin B_2_ or FAD. The photodegradation of the drug in D_2_O was monitored by ^1^H nuclear magnetic resonance spectroscopy.

**Results:**

Dacarbazine dissolved in D_2_O decomposed in the presence of vitamin B_2_ or FAD under irradiation with LED or fluorescent light. The decomposition products were 2-azahypoxanthine **2**, which has previously been observed after light irradiation in the absence of vitamin B_2_, and 1*H*-imidazole-5-carboxamide **6**, a new product formed in the presence of vitamin B_2_. Irradiation with LED light was more effective than irradiation with fluorescent light in degrading dacarbazine.

**Conclusion:**

Vitamin B_2_ and FAD induced dacarbazine photodegradation. Thus, the interfusion of vitamin B_2_ or FAD under excessive light exposure should be avoided during the intravenous administration of dacarbazine.

**Graphical Abstract:**

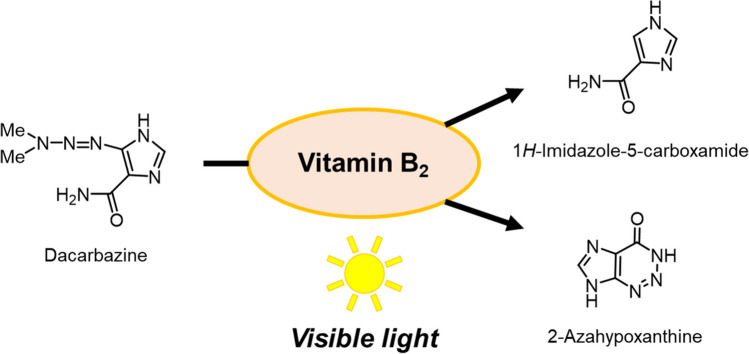

**Supplementary Information:**

The online version contains supplementary material available at 10.1007/s11095-024-03802-2.

## Introduction

**Scheme 1:**
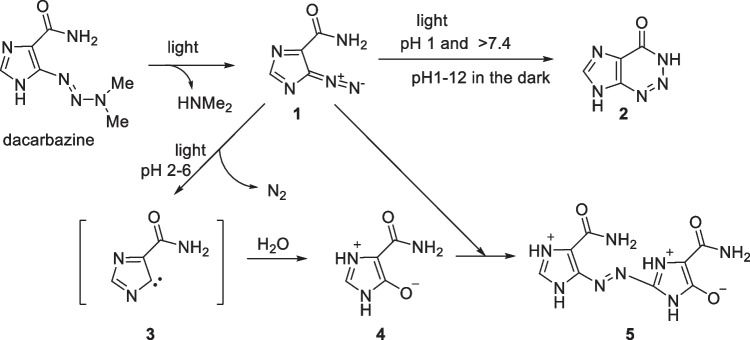
Process of dacarbazine degradation under light irradiation.

Dacarbazine (Scheme 1) is an anticancer drug used to treat Hodgkin’s disease, malignant melanoma [1], soft-tissue sarcoma [2], and childhood solid tumors [3]. When metabolized, dacarbazine produces diazomethane, which alkylates the nucleic acids of tumor cells and exerts antitumor effects [4]. Aqueous solutions of this drug turn pink when exposed to light because of the formation of degradation products. After a pioneering study on dacarbazine degradation by Shealy [5, 6], Horton reported that the photodegradation of dacarbazine was influenced by the environmental pH, and described the degradation process in detail (Scheme 1). Dacarbazine decomposes under light conditions to produce dimethylamine and 5-diazoimidazole-4-carboxamide **1**. Because **1** is highly photoreactive, different reactions proceed under light or different pH conditions to give 2-azahypoxanthine** 2**, 4-carbamoylimidazolium-5-olate **4**, and azoimidazole **5**, which is the coupling compound of **1** and** 4** [7].

However, subsequent studies reported that only **2** was obtained from **1** under similar conditions [8–10], which contradicted the study of Horton.

**Fig. 1:**
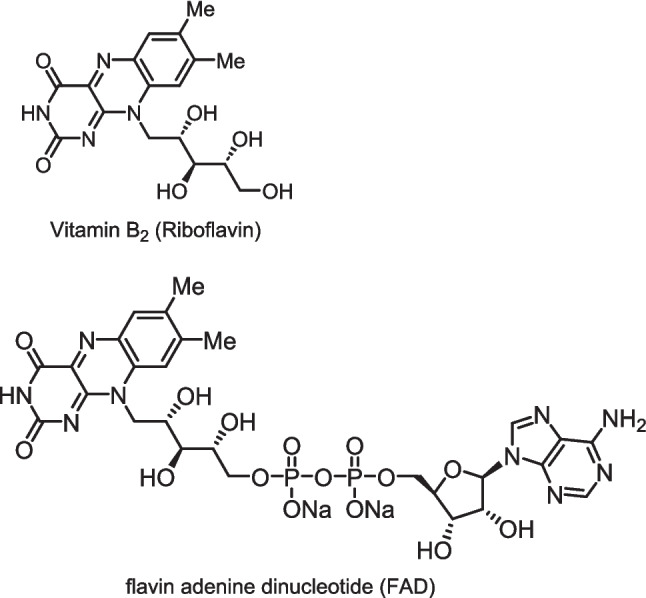
Chemical structures of vitamin B_2_ and flavin adenine dinucleotide (FAD).

Riboflavin, also known as vitamin B_2_ (Fig. 1), was recently reported to be an efficient photosensitizer for *E*/*Z* isomerization reactions [11]. Considering that such isomerization reactions may occur in the clinical setting, we investigated the possibility of the photoisomerization of the double-bond structures of sulindac and ozagrel hydrochloride in the presence of vitamin B_2_ or flavin adenine dinucleotide (FAD) (Fig. 1) under light irradiation. The photoisomerization of the drugs occurred in D_2_O under both 405 nm light-emitting diode (LED) and fluorescent light irradiation, although the process was slower in the presence of fluorescent light [12]. McNeill reported that amino acids were degraded in the presence of vitamin B_2_ and its derivatives under visible-light irradiation [13].

Vitamin B_2_ or FAD can be administered as an ingredient of high-calorie infusions. However, their concomitant administration with other intravenous drugs, such as dacarbazine, may induce photodegradation. To the best of our knowledge, no study on the degradative effects of these compounds in the presence of light irradiation has yet been reported. To confirm whether such photodegradation occurs, we investigated the photodegradation of dacarbazine in the presence of vitamin B_2_ or FAD under LED or fluorescent light irradiation.

## Materials and Methods

### Materials

Dacarbazine and FAD were obtained from Tokyo Chemical Industry (Tokyo, Japan). Vitamin B_2_ and D_2_O were purchased from Wako Pure Chemical Industries (Osaka, Japan).

### Photodegradation Under Light

A nuclear magnetic resonance (NMR) tube (ϕ: 4.95 mm) containing a solution of dacarbazine (5.0 × 10^–3^ M in D_2_O) was added with 0.01, 0.05, 0.5, or 1.0 equivalent of vitamin B_2_. For LED light (405 nm; 18 W) irradiation, the tube was set on an optical irradiation apparatus (EvoluChem PhotoRedOx Box™ and chemistry screening kits; HCK1006-01–016, HCK1012-01–010; HepatoChem Inc., MA, USA) at a distance of 5 cm from the light source and irradiated with a LED light at 25°C for a fixed time. For fluorescent light (40 W, SL-202; Nitori Co., Ltd, Japan) irradiation, the tube was set at a distance of 10 cm from the light source and irradiated with a fluorescent light at 25°C for a fixed time. The reaction progress was monitored via ^1^H NMR spectroscopy using an NMR spectrometer (JEOL GX-400; JMN-ECZ400S; JEOL Ltd., Tokyo, Japan) at 400 MHz (Delay time: 5 s; pulse angle: 45°; offset: 5 ppm; spectrum width: 7.949 kHz; acquisition time: 2 s; ^13^C decoupling: OFF; number of scans: 16 times; dummy scan: one time; and temperature control: OFF) and at 296 K. Chemical shifts are reported in ppm (δ) relative to residual D_2_O (δ 4.79) as an internal reference.

### Purification of Degradation Products 2 and 6

Dacarbazine (60 mg, 0.329 mmol) in water (5.0 × 10^–3^ M) and 0.05 equivalent of vitamin B_2_ were added to the vessel. The reaction mixture was irradiated with LED light from a light source placed at a distance of 5 cm from the mixture at 25 ℃ for 1.5 h. After irradiation, the reaction mixture was concentrated *in vacuo*. The concentrate was purified by column chromatography (silica gel, CH_2_Cl_2_/MeOH/H_2_O = 24:7:1) to afford compounds **2** (14.7 mg, 0.106 mmol, 32%) and 1*H*-imidazole-5-carboxamide **6** (15.1 mg, 0.136 mmol, 41%) as pale yellow solids.

- 1*H*-Imidazole-5-carboxamide **6**
- ^1^H NMR (400 MHz, D_2_O): δ 7.75 (s, 1H), 7.78 (s, 1H); ^13^C NMR (DMSO-*d*_6_): δ 119.2, 135.5, 136.5, 164.4; HRMS (ESI-TOF): m/z [M+H]^+^calcd for C_4_H_6_N_3_O: 112.0505; found: 112.0505

## Results and Discussion

### Photodegradation of Dacarbazine under 405 nm LED Light Irradiation

In general, photochemical degradation is identified using high-performance liquid chromatography–mass spectrometry [14–20]. In this study, dacarbazine photodegradation was monitored by ^1^H NMR spectroscopy, which allows for the simultaneous determination of the structures of the photodegradation products. First, the ^1^H NMR spectrum of dacarbazine in D_2_O was recorded.

**Fig. 2:**
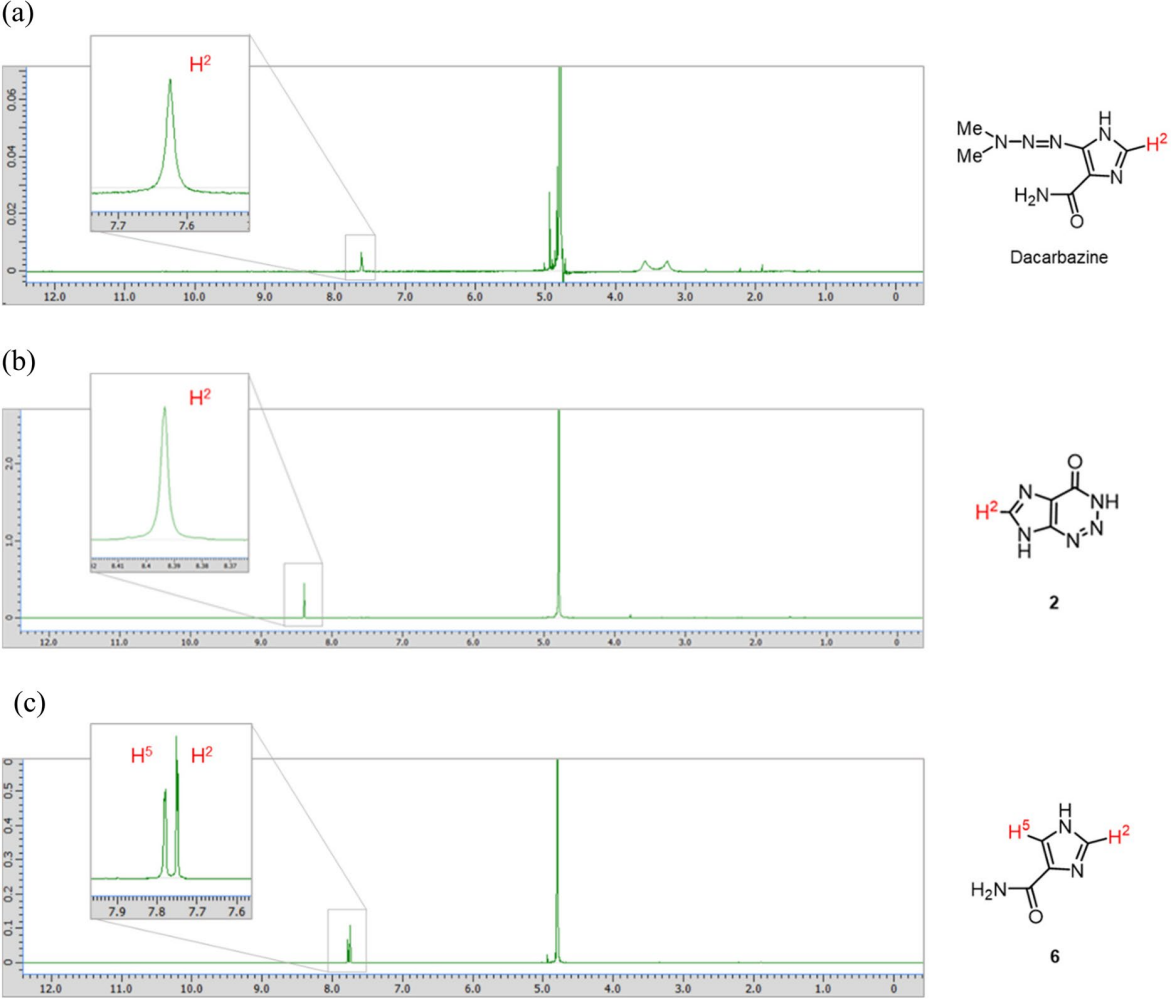
Nuclear magnetic resonance spectra of (**a**) dacarbazine in D_2_O, (**b**) 2-azahypoxanthine (**2**), and (**c**) 1*H*-imidazole-5-carboxamide (**6**).

**Fig. 3:**
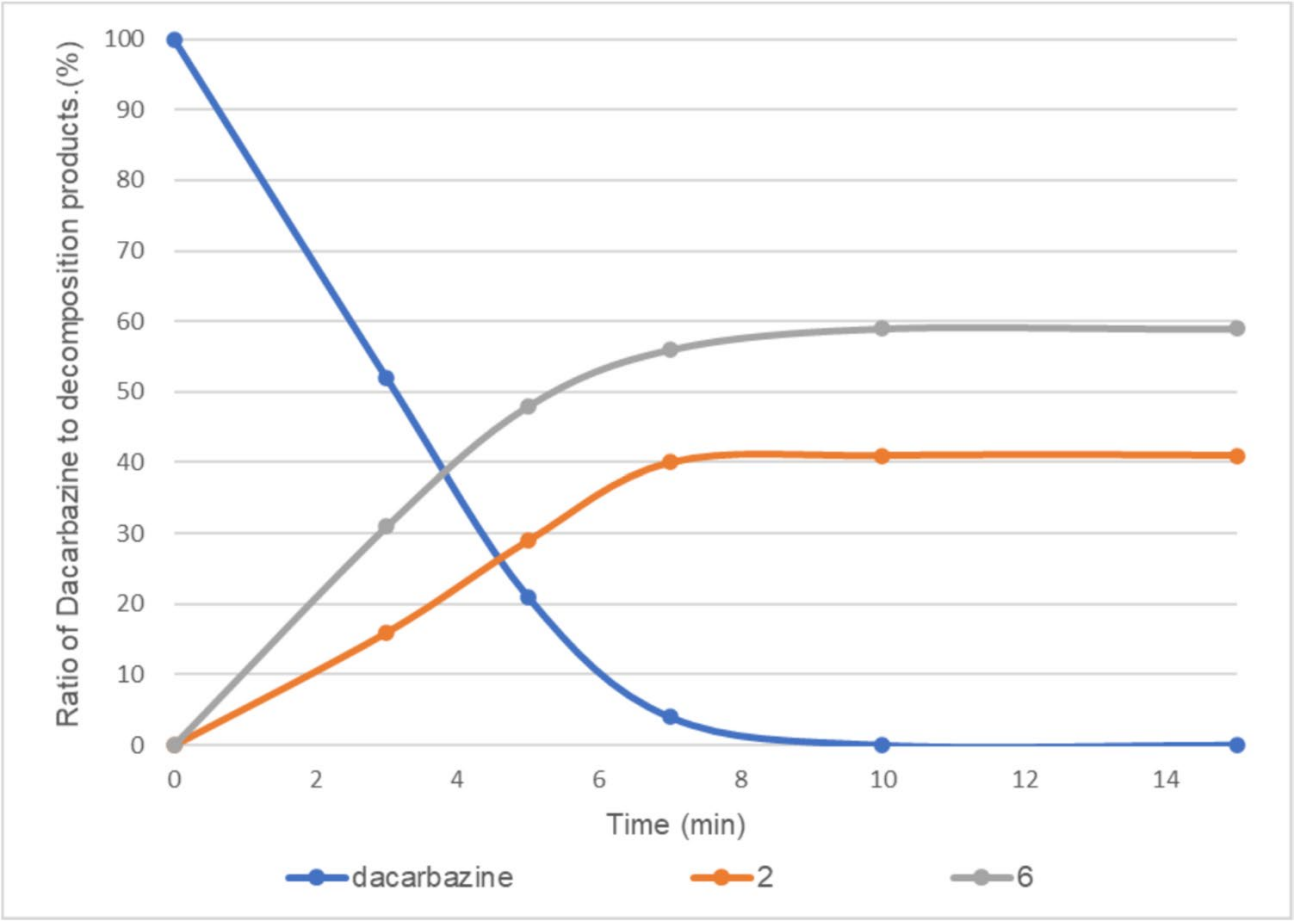
Light-emitting diode light (405 nm)-induced photodegradation of dacarbazine in D_2_O (5.0 mM) in the presence of 0.05 equivalent of vitamin B_2_. **2**: 2-azahypoxanthine, **6**: 1*H*-imidazole-5-carboxamide. The line represents the connecting of the data points.

The H-2 proton of the imidazole moiety of dacarbazine was observed at approximately 7.62 ppm, which is a characteristic peak confirming the presence of dacarbazine (Fig. 2a). Next, 0.05 equivalent of vitamin B_2_ was added to dacarbazine dissolved in D_2_O. The mixture was irradiated with LED light, which is the absorption wavelength of vitamin B_2_, and the progress of the photodegradation reaction was monitored by ^1^H NMR spectroscopy. After 10 min of irradiation, dacarbazine completely disappeared from the reaction system, and the two degradation compounds were observed at a ratio of approximately 40:60 (Fig. 3). After purification by column chromatography, compound **2** (Fig. 2b) was isolated as a by-product and compound **6** [21] (Fig. 2c) was isolated as the major product. Notably, **6** was obtained for the first time as a degradation product of dacarbazine.

**Fig. 4:**
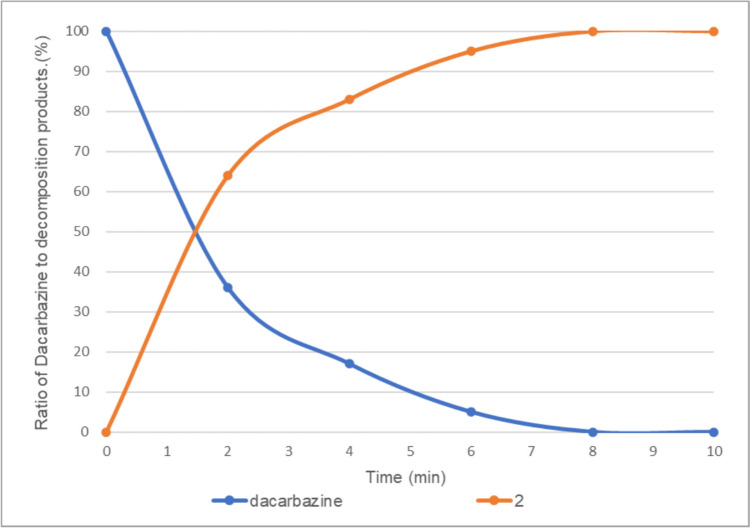
Light-emitting diode light (405 nm)-induced photodegradation of dacarbazine in D_2_O (5.0 mM) without vitamin B_2_. **2**: 2-azahypoxanthine. The line represents the connecting of the data points.

By contrast, irradiation with LED light for 8 min in the absence of vitamin B_2_ completely decomposed dacarbazine to provide **2** as the sole product (Fig. 4). In this study, compounds **1**, **4**, and **5**, which are shown in Scheme 1, were not obtained, regardless of the presence or absence of vitamin B_2_.

Based on these results, we presumed that compound **6** was produced by the coexistence of vitamin B_2_ and dacarbazine. Therefore, we investigated the effect of light irradiation on dacarbazine mixed with varying amounts of vitamin B_2_.

**Fig. 5:**
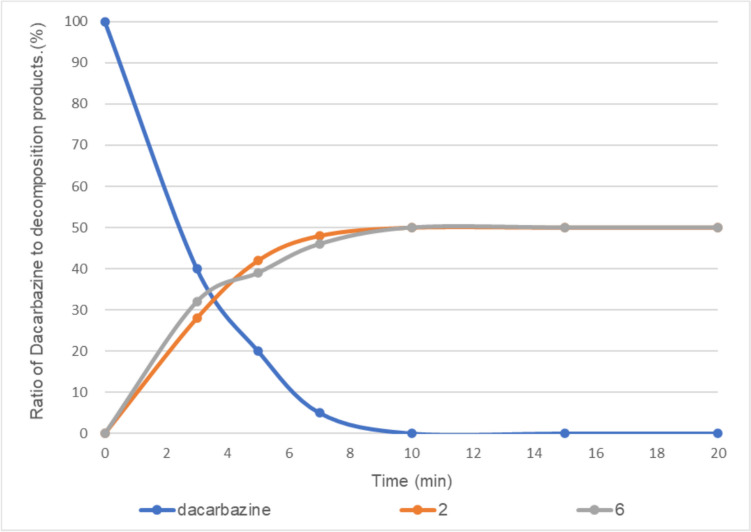
Light-emitting diode light (405 nm)-induced photodegradation of dacarbazine in D_2_O (5.0 mM) in the presence of 0.01 equivalent of vitamin B_2_. **2**: 2-azahypoxanthine, **6**: 1*H*-imidazole-5-carboxamide. The line represents the connecting of the data points.

In the presence of 0.01 equivalent of vitamin B_2_, dacarbazine disappeared after 10 min of irradiation to provide compounds **2** and **6** at a ratio of approximately 50:50 (Fig. 5).

**Fig. 6:**
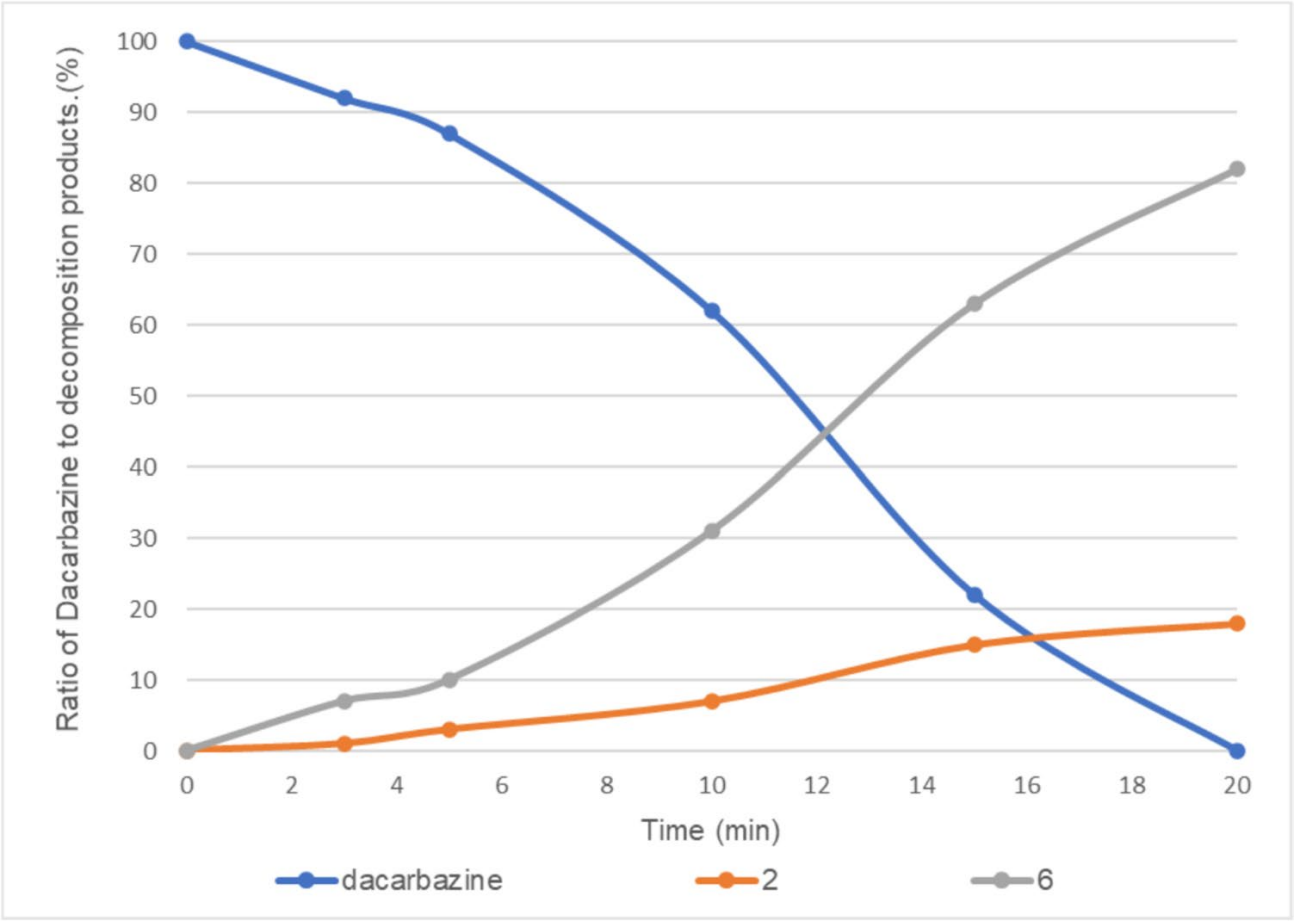
Light-emitting diode light (405 nm)-induced photodegradation of dacarbazine in D_2_O (5.0 mM) in the presence of 0.5 equivalent of vitamin B_2_. **2**: 2-azahypoxanthine, **6**: 1*H*-imidazole-5-carboxamide. The line represents the connecting of the data points.

**Fig. 7:**
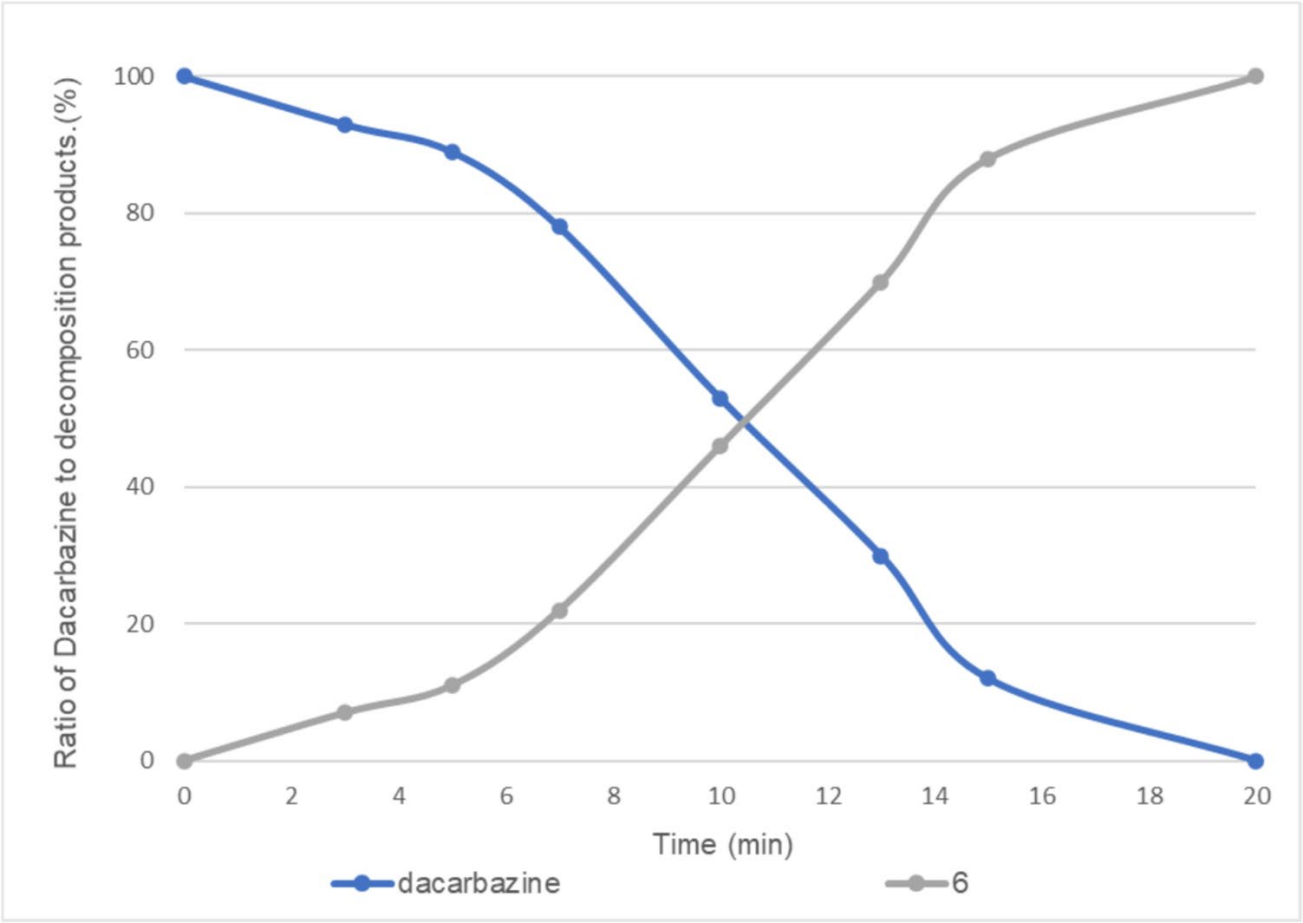
Light-emitting diode light (405 nm)-induced photodegradation of dacarbazine in D_2_O (5.0 mM) in the presence of 1.0 equivalent of vitamin B_2_. **6**: 1*H*-imidazole-5-carboxamide. The line represents the connecting of the data points.

In the presence of 0.5 equivalent of vitamin B_2_, dacarbazine disappeared after 20 min of irradiation, and compounds **2** and **6** were observed at a ratio of approximately 19:81 (Fig. 6). When the amount of vitamin B_2_ was further increased to 1 equivalent, dacarbazine disappeared after 20 min of light irradiation, and compound **6** was obtained as the sole product (Fig. 7).

Table I summarizes the results shown in Figs. 3, 4, 5, 6, and 7. As the amount of vitamin B_2_ increased, the amount of compound **2** decreased whereas the amount of compound **6** increased. Therefore, compound **6** may be obtained through a reaction mechanism different from that of compound **2** owing to the influence of vitamin B_2_.

**Table I:** Ratio of 2-azahypoxanthine **2** and 1*H*-imidazole-5-carboxamide **6** Obtained under Different Amounts of Vitamin B_2_

Entry	Vitamin B_2_ (equiv.)	**2** (%)	**6** (%)
1	0	100	0
2	0.01	50	50
3	0.05	40	60
4	0.5	19	81
5	1.0	0	100

**Fig. 8:**
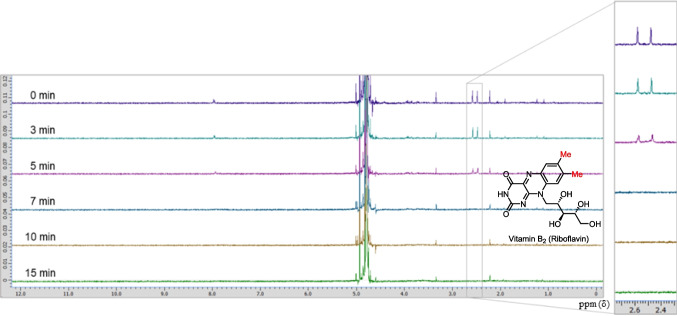
Nuclear magnetic resonance spectra of the light-emitting diode light (405 nm)-induced changes in vitamin B_2_ (0.3 μmol) in D_2_O (0.25 mM).

The NMR experiments demonstrated that vitamin B_2_ was decomposed by LED light irradiation. We dissolved vitamin B_2_ in D_2_O, irradiated the solution with 405 nm LED light, and monitored the progress of the photodegradation reaction by ^1^H NMR spectroscopy (Fig. 8). Two methyl groups substituted on the benzene ring were observed at approximately 2.48 and 2.58 ppm, which are characteristic peaks confirming the presence of vitamin B_2_. As the irradiation time increased, the intensity of these peaks decreased and disappeared completely after 7 min of irradiation. The NMR spectra also showed that many degradation products were produced along with the decomposition of vitamin B_2_.

**Fig. 9:**
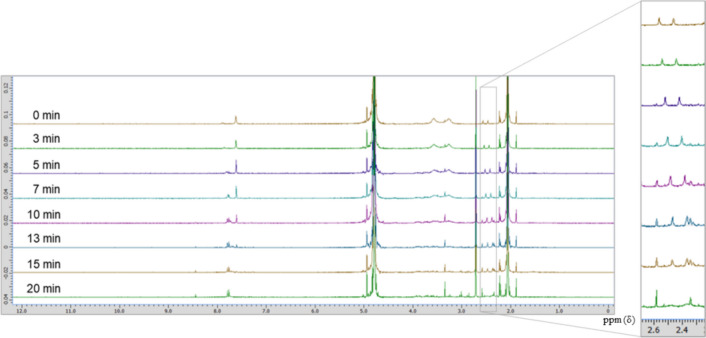
Nuclear magnetic resonance spectra of the light-emitting diode light (405 nm)-induced photodegradation of dacarbazine in D_2_O (5.0 mM) in the presence of 1.0 equivalent of vitamin B_2_.

The progress of the photolysis of an equimolar mixture of dacarbazine and vitamin B_2_ was measured by NMR spectroscopy (Fig. 9). The characteristic methyl peaks of vitamin B_2_ changed into different methyl peaks over 20 min of irradiation. Compound **6** was produced simultaneously. Based on these results, we speculated that vitamin B_2_ is excited by light irradiation and reacts with dacarbazine to produce compound **6**. Unfortunately, owing to the large number of degradation products obtained, no degradation products of vitamin B_2_ were identified in this experiment. Therefore, we could not elucidate the mechanism by which dacarbazine reacts with excited vitamin B_2_ to produce compound **6**.

### Photodegradation of Dacarbazine in the Presence of Flavin Adenine Dinucleotide (FAD)

**Fig. 10:**
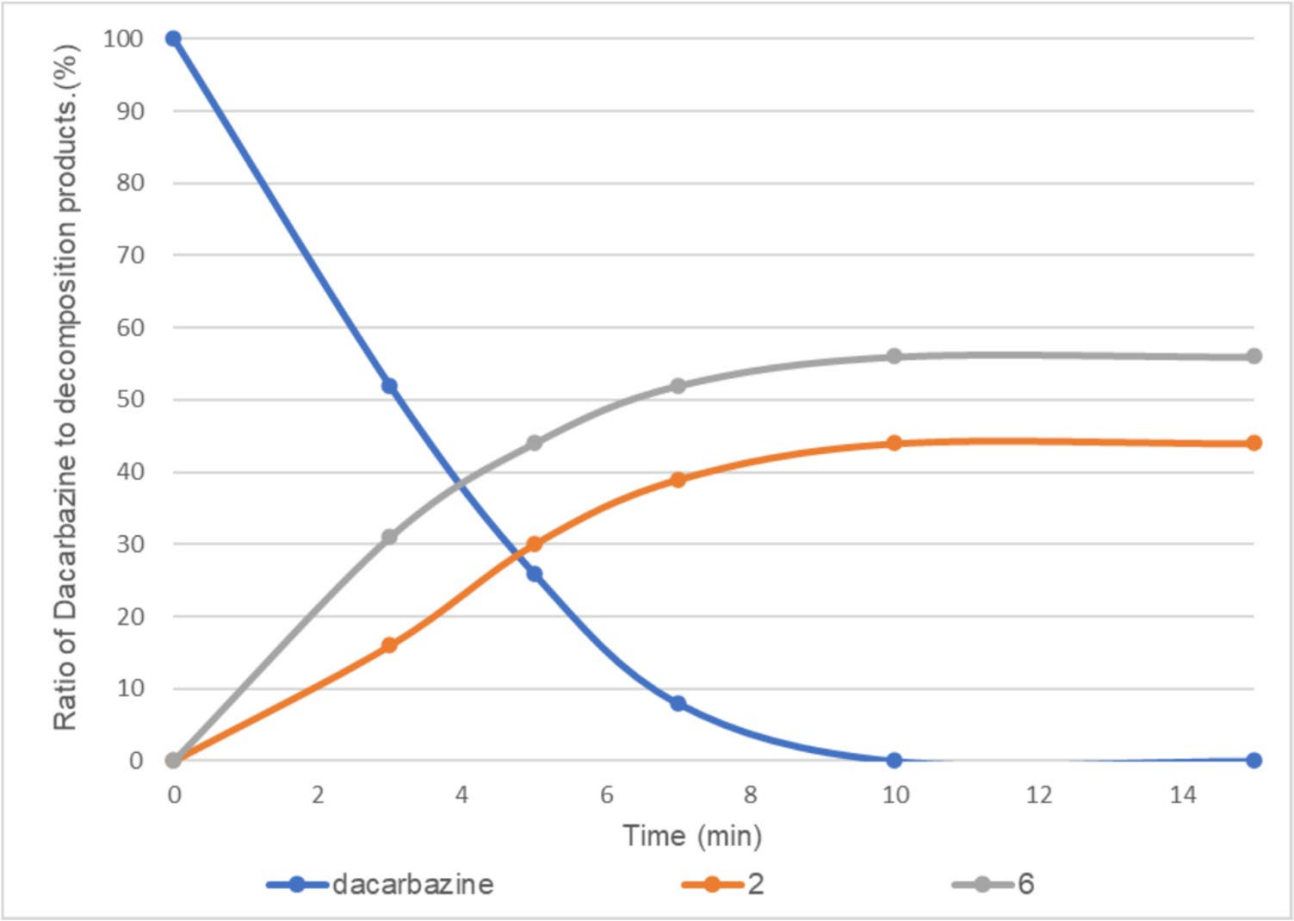
Light-emitting diode light (405 nm)-induced photodegradation of dacarbazine in D_2_O (5.0 mM) in the presence of flavin adenine dinucleotide. **2**: 2-azahypoxanthine, **6**: 1*H*-imidazole-5-carboxamide. The line represents the connecting of the data points.

Next, we examined the effect of FAD (Fig. 1) on the photodegradation of dacarbazine. FAD, a coenzyme, is also present in high-calorie infusions. Because the structure of FAD is similar to that of vitamin B_2_, we anticipated that FAD would also function as a photosensitizer to promote dacarbazine degradation. Therefore, we investigated the photolysis of dacarbazine with 0.05 equivalent of FAD under 405 nm LED light irradiation. After 10 min, dacarbazine completely disappeared, and the degradation compounds **2** and** 6** were observed at a ratio of approximately 45:55. (Fig. 10). Thus, the results of dacarbazine degradation in the presence of FAD and vitamin B_2_ are similar (Fig. 3).

### Photodegradation of Dacarbazine under Fluorescent Light

**Fig. 11:**
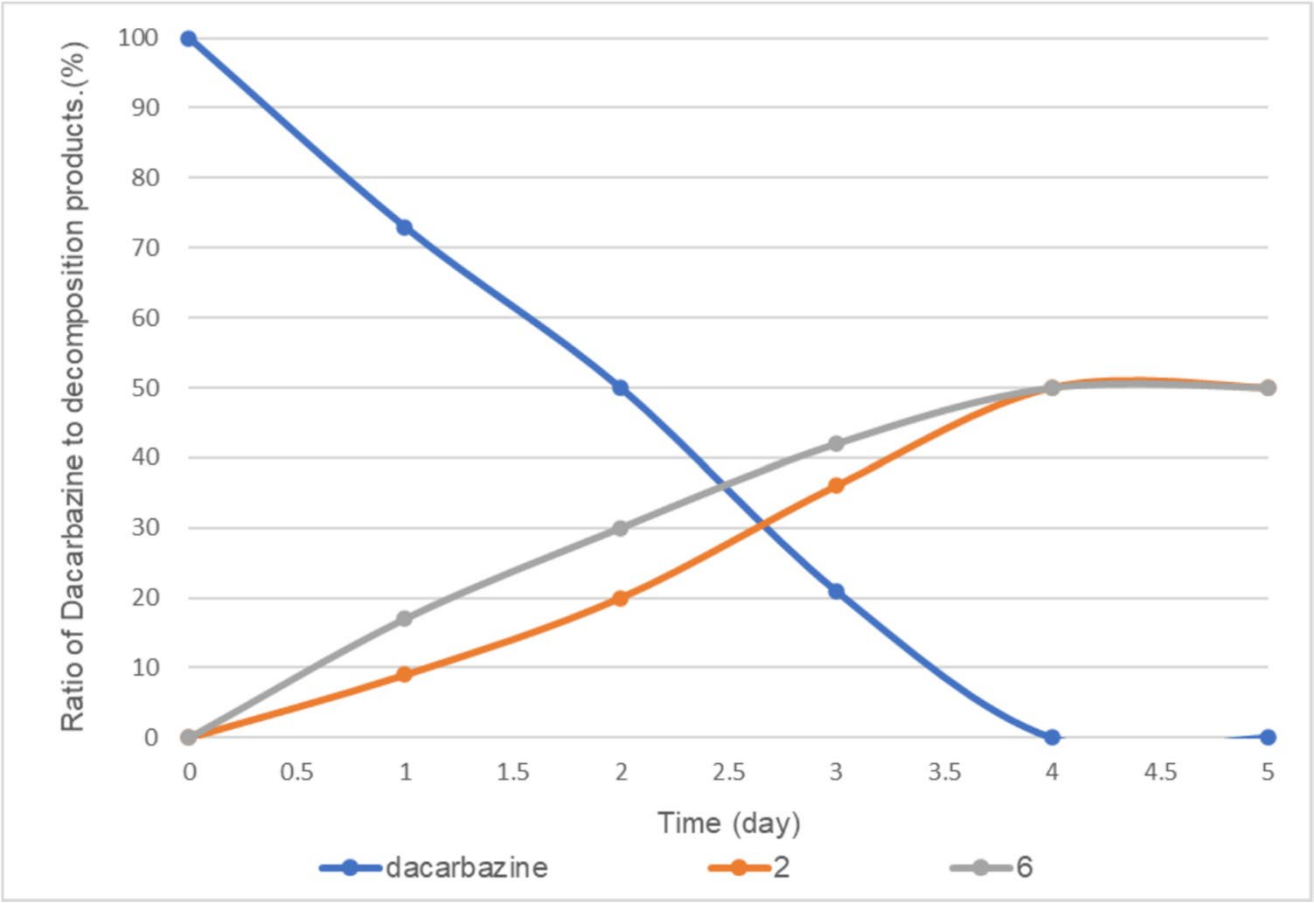
Fluorescent light-induced photodegradation of dacarbazine in D_2_O (5.0 mM) in the presence of 0.05 equivalent of vitamin B_2_. **2**: 2-azahypoxanthine, **6**: 1*H*-imidazole-5-carboxamide. The line represents the connecting of the data points.

Next, the photodegradation of dacarbazine under fluorescent light (400–700 nm) was examined. In this case, dacarbazine was degraded very slowly because of the low energy of fluorescent light. When 0.05 equivalent of vitamin B_2_ was added to dacarbazine, dacarbazine completely disappeared from the reaction system over 4 days, and degradation compounds **2** and **6** were observed at a ratio of approximately 50:50 (Fig. 11). The slight difference between the ratios of **2** and **6** under LED (405 nm) and fluorescent light irradiation may be attributed to the various wavelengths present in fluorescence light.

**Fig. 12:**
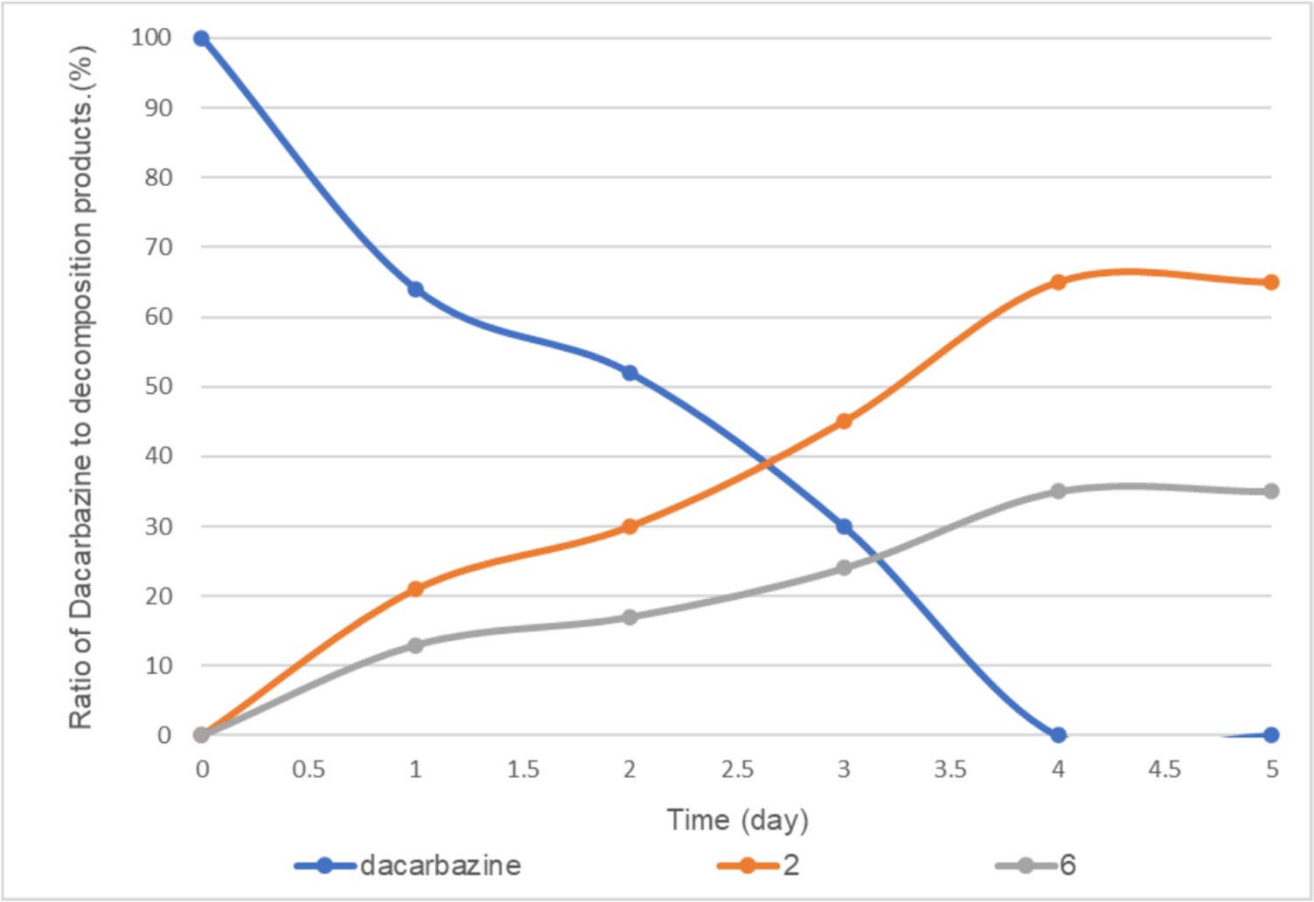
Fluorescent light-induced photodegradation of dacarbazine in D_2_O (5.0 mM) in the presence of 0.01 equivalent of vitamin B_2_. **2**: 2-azahypoxanthine, **6**: 1*H*-imidazole-5-carboxamide. The line represents the connecting of the data points.

When the amount of vitamin B_2_ was reduced to 0.01 equivalent, dacarbazine disappeared completely after 4 days, and compounds **2** and **6** were observed at a ratio of approximately 65:35 (Fig. 12). Similar to the case with LED light irradiation, the amount of vitamin B_2_ affected the ratio of degradation products **2** and **6** with fluorescent light irradiation. Notably, the amount of compound **2** produced under fluorescent light irradiation was greater than that produced under LED light irradiation.

Fluorescent light is emitted at wavelengths between 400 and 700 nm, and the absorption wavelength of vitamin B_2_ is 405 nm. Therefore, we can postulate that vitamin B_2_ produces compound **6** even under low-energy fluorescent light.

**Fig. 13:**
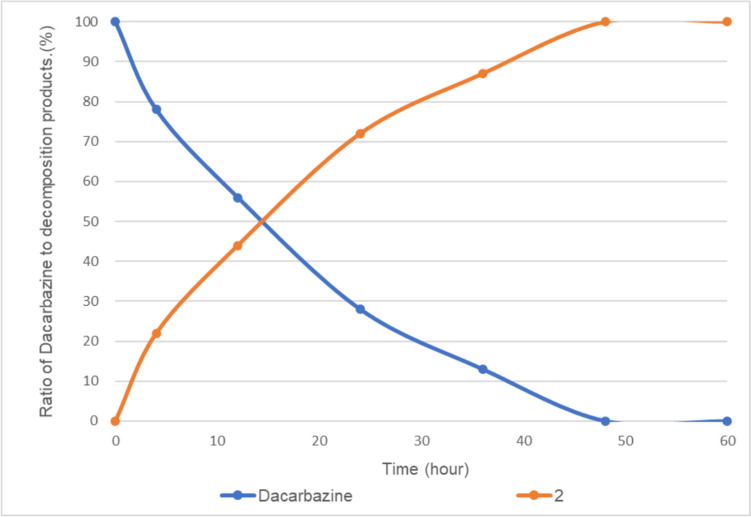
Fluorescent light-induced photodegradation of dacarbazine in D_2_O (5.0 mM) in the absence of vitamin B_2_. The line represents the connecting of the data points.

Additionally, we investigated the degradation products obtained upon exposing dacarbazine to fluorescent light in the absence of vitamin B_2_. The results were the same as those reported previously [8–10], with only compound **2** being obtained as a degradation product (Fig. 13).

### Determination of the Kinetic Parameters of Photodegradation

**Fig. 14:**
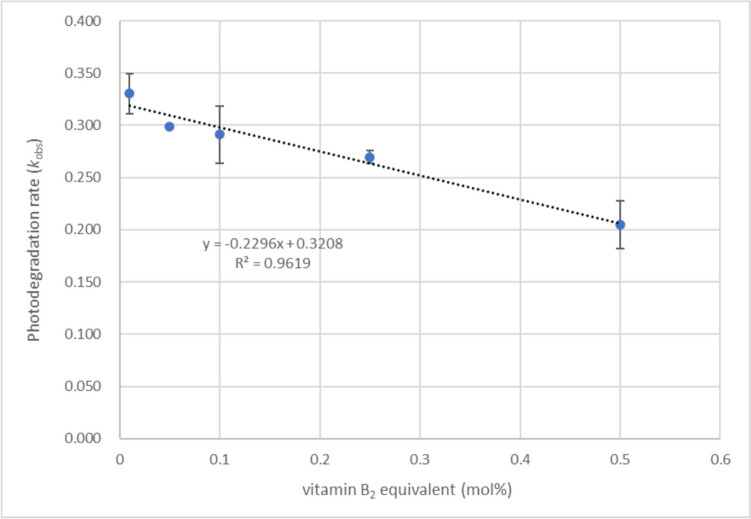
First-order dependence of the dacarbazine degradation rate on the amount of vitamin B_2_ added to the reaction system. Error bars represent standard deviations of triplicates.

Finally, the kinetics of dacarbazine photodegradation was investigated. The degradation reaction followed first-order kinetics. We simplify fitting the initial rates. The concentration of dacarbazine calculated from the NMR spectra was plotted against time, and *t*_1/2_ was calculated from the resulting plot to obtain the pseudo-first-order rate constant *k*_obs_ and the second-order rate constant *k*_2_ (Table II). Changes in the photodegradation rate of dacarbazine under various vitamin B_2_ concentrations are shown in Fig. 14. The *t*_1/2_ value of the degradation of dacarbazine under 405 nm LED light irradiation in the presence of a catalytic amount of vitamin B_2_ was calculated to be 2.5 min. Similarly, the *t*_1/2_ value of the degradation of dacarbazine under 405 nm LED light irradiation in the presence of FAD was calculated to be 2.9 min. The photodegradation of dacarbazine proceeded more rapidly in the absence of a photosensitizer (*t*_1/2_ = 1.6). These results suggest that the photosensitizer, which absorbs light, reduces the light irradiation of dacarbazine, thereby decreasing its decomposition rate. The reaction induced by the photosensitizer may also be slower than the decomposition reaction that occurs when dacarbazine is directly irradiated with light. The degradation reaction was much slower under fluorescent light irradiation than under LED light irradiation, and the decrease in reaction rate under fluorescent light irradiation was observed in all cases (Tables II, III, and IV). Because the energy of fluorescent light is low, the photoreaction proceeds slowly.

**Table II:** Rate Constants of the Photodegradation of Dacarbazine

Light source	Solvent	Photosensitizer (equivalent)	*k*_obs_ (min^−1^)	*k*_2_ (M^−1^ min^−1^)	*t*_1/2_ (min)
LED light (405 nm)	D_2_O	Vitamin B_2_ (0.05)	285 × 10^–3^	114 × 10^–2^	2.5
		FAD (0.05)	237 × 10^–3^	95 × 10^–2^	2.9
		No photosensitizer	443 × 10^–3^		1.6
Fluorescent light	D_2_O	Vitamin B_2_ (0.05)	0.35 × 10^–3^	0.14 × 10^–2^	1981.4
		Vitamin B_2_ (0.01)	0.54 × 10^–3^	0.22 × 10^–2^	1288.9

**Table III:** Rate Constants of the Photoproduction of Compound **2**

Light source	Solvent	Photosensitizer (equivalent)	*k*_obs_ (min^−1^)	*k*_2_ (M^−1^ min^−1^)	*t*_1/2_ (min)
LED light (405 nm)	D_2_O	Vitamin B_2_ (0.05)	181 × 10^–3^	72 × 10^–2^	3.8
		FAD (0.05)	176 × 10^–3^	71 × 10^–2^	3.9
		No photosensitizer	463 × 10^–3^		1.5
Fluorescent light	D_2_O	Vitamin B_2_ (0.05)	0.23 × 10^–3^	0.09 × 10^–2^	3078.0
		Vitamin B_2_ (0.01)	0.48 × 10^–3^	0.19 × 10^–2^	1434.8

**Table IV:** Rate Constants of the Photoproduction of Compound **6**

Light source	Solvent	Photosensitizer (equivalent)	*k*_obs_ (min^−1^)	*k*_2_ (M^−1^ min^−1^)	*t*_1/2_ (min)
LED light (405 nm)	D_2_O	Vitamin B_2_ (0.05)	270 × 10^–3^	108 × 10^–2^	2.6
		FAD (0.05)	306 × 10^–3^	122 × 10^–2^	2.3
Fluorescent light	D_2_O	Vitamin B_2_ (0.05)	0.29 × 10^–3^	0.12 × 10^–2^	2372.6
		Vitamin B_2_ (0.01)	0.34 × 10^–3^	0.14 × 10^–2^	2043.0

The concentrations of compounds **2** and **6** calculated from the NMR spectra were plotted as a function of time, and the *t*_1/2_ values of their formation reaction were calculated from the resulting plots to obtain the corresponding *k*_obs_ and *k*_2_ values (Tables III and IV). The *t*_1/2_ value of the formation of compound **2** under LED light irradiation in the presence of catalytic amounts of vitamin B_2_ and FAD was 3.8 min. Even in this case, the rate of formation of compound **2** was higher in the absence of a photosensitizer than in its presence. The *t*_1/2_ values of the formation of compound **6** under LED light irradiation in the presence of catalytic amounts of vitamin B_2_ and FAD were 3.8 min and 3.9 min, respectively. Interestingly, the formation rates of compounds **2** and **6** were similar.

**Scheme 2:**
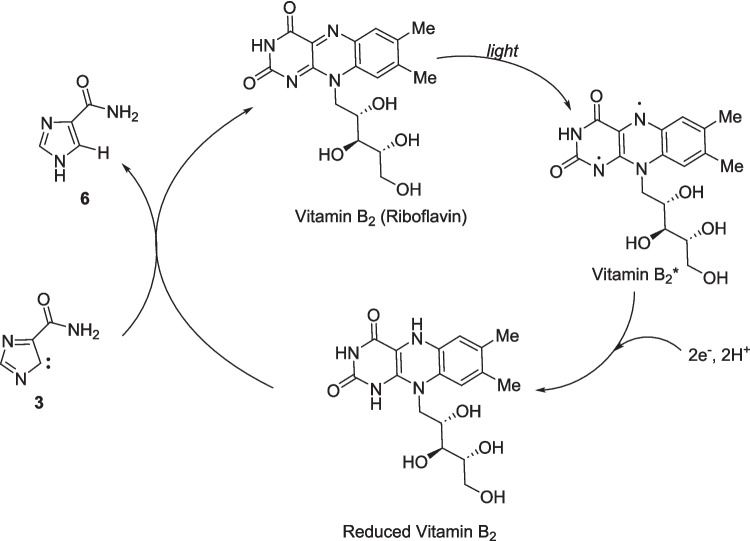
Proposed mechanism.

Notably, compound **6** has been obtained for the first time in the presence of vitamin B_2_. The formation of compound **6** is assumed to proceed through formal reduction of the carbene intermediate **3**. The available information is limited, and using D_2_O as a solvent does not lead to deuterium incorporation. Nevertheless, we hypothesize that vitamin B_2_ should be involved in this step as a reducing agent. A proposed reaction mechanism is illustrated in Scheme 2.

Under visible-light excitation, a flavin molecule (Vitamin B_2_*) reduces (reduced Vitamin B_2_) by gaining two electrons and two protons from the substrate or its degraded products. The reduced vitamin B_2_ donates a hydride and proton to compound **3**, which then converts to compound **6**, and simultaneously returns to its ground state (Vitamin B_2_).

The results revealed that the photodegradation of dacarbazine in D_2_O under fluorescent light proceeds very slowly. Considering that such a slow reaction requires a longer reaction time to reach completion, photodegradation is unlikely to occur during the very short mixing time in the intravenous line. In the clinical setting, injectable drugs are administered in parallel or mixed with a nutritional infusion from a side tube. Because the mixing time in the line is limited, the degree of photodegradation may be negligible. Nevertheless, this study suggests the importance of considering that mixing vitamin B_2_ or FAD with dacarbazine leads to a decrease in drug potency.

## Conclusions

Irradiation of a mixture of dacarbazine and vitamin B_2_ or FAD with 405 nm LED or fluorescent light generated a new degradation product, 1*H*-imidazole-5-carboxamide **6**, in addition to 2-azahypoxanthine **2**, the previously reported degradation product of dacarbazine. The amount of compound **6** produced during the degradation reaction depended on the amount of vitamin B_2_ or FAD added to the reaction system. Based on the NMR measurements, vitamin B_2_ is excited by light irradiation and reacts with dacarbazine to produce compound **6**. Although the dacarbazine degradation rate under fluorescent light was remarkably slower than that under LED light, dacarbazine degradation occurred in the presence of both vitamin B_2_ and FAD. This study confirmed that the combination of vitamin B_2_ or FAD with dacarbazine reduced the efficacy of dacarbazine. Therefore, excessive light exposure should be avoided during the intravenous administration of dacarbazine with possible vitamin B_2_ or FAD contamination. We suggest that the addition of vitamin B_2_ or FAD results in new photodegradation reactions that have not been previously observed in the absence of these substances. Thus, vitamins B_2_ and FAD, which act as photosensitizers, must be handled carefully. Furthermore, other drugs that act as photosensitizers may also induce photodegradation reactions. We are currently investigating such decomposition reactions and will report our findings in due course.

## Supplementary Information

Below is the link to the electronic supplementary material.

Supplementary file1 (PDF 574 KB)
