# Supplementary material for: Photodegradation of Dacarbazine Catalyzed by Vitamin B2 and Flavin Adenine Dinucleotide Under Visible-Light Irradiation
Source: Pharm Res. 2024 Dec 18;41(12):2363–75. doi: 10.1007/s11095-024-03802-2 (PMC11682012; doi:10.1007/s11095-024-03802-2)
Supplement: Supplementary file 1 — Supplementary file1 (PDF 574 KB) [file 11095_2024_3802_MOESM1_ESM.pdf]

Supporting Information

Photodegradation of Dacarbazine Catalyzed by Vitamin B2 and Flavin Adenine Dinucleotide Under Visible-Light Irradiation

Yuka Kimura, Mayuko Suga, Kayo Nakamura, Hidetsugu Tabata, Tetsuta Oshirari, Hideaki Natsugari, and Hideyo Takahashi

*Corresponding Author: hide-tak@rs.tus.ac.jp

Contents

1. **Identification of compound 6………..……….…………………………..…..S3**

2. **Determination of the kinetic parameters of photoisomerization ……………S4**

1. Identification of compound 6

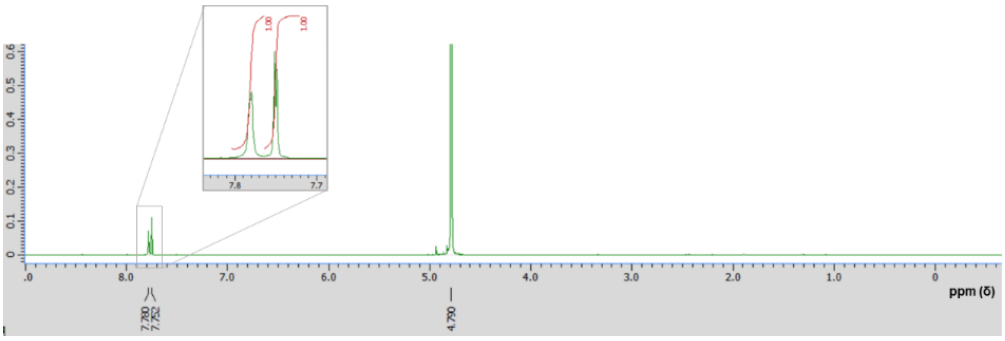

Figure S1 ^{1}H NMR (400 MHz, D_2O) spectra of **6**.

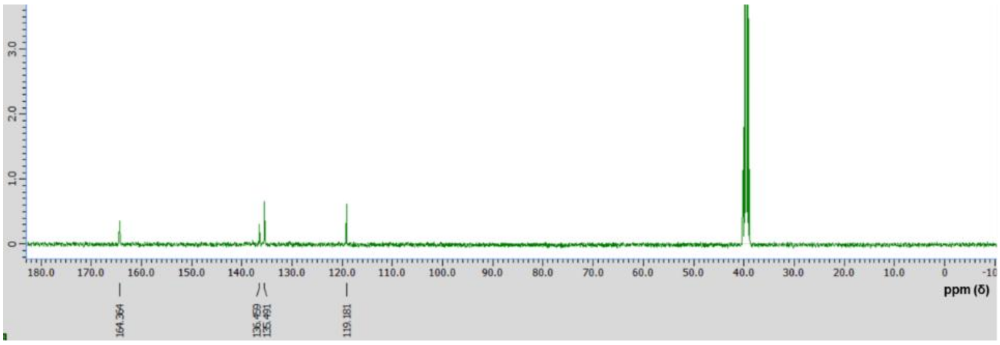

Figure S2 ^{13}C NMR (400 MHz, DMSO-d_6) spectra of **6**.

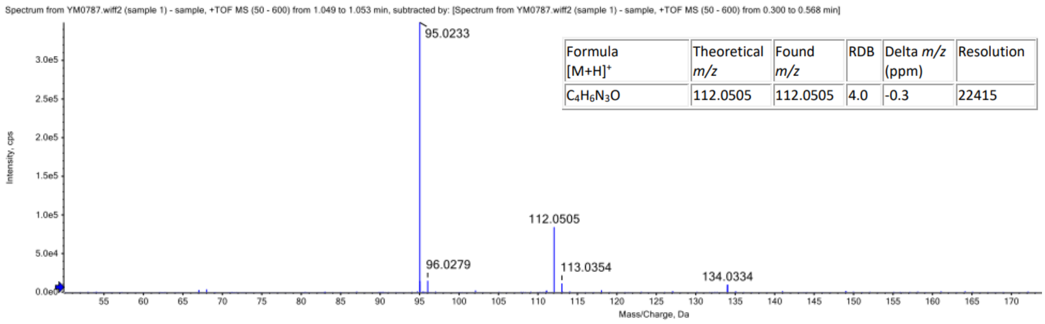

Formula $[M+H]^+$	Theoretical *m/z*	Found *m/z*	RDB	Delta *m/z* (ppm)	Resolution
$C_4H_6N_3O$	112.0505	112.0505	4.0	-0.3	22415

Fig. S3 HRMS (ESI-TOF) spectra of **6**.

2. Determination of the kinetic parameters of photoisomerization

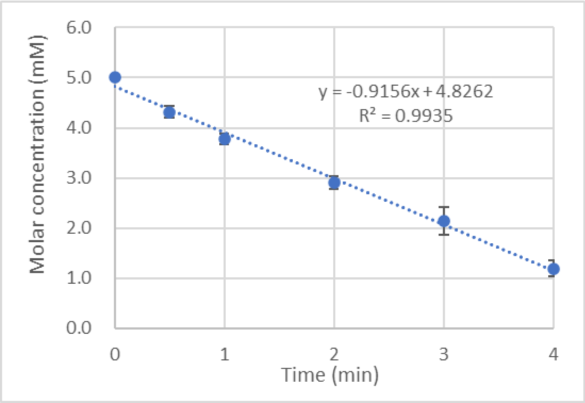

Fig. S4 Time-dependent concentration changes of dacarbazine in the presence of 0.05 equivalents of riboflavin. Error bars represent standard deviations of triplicates.

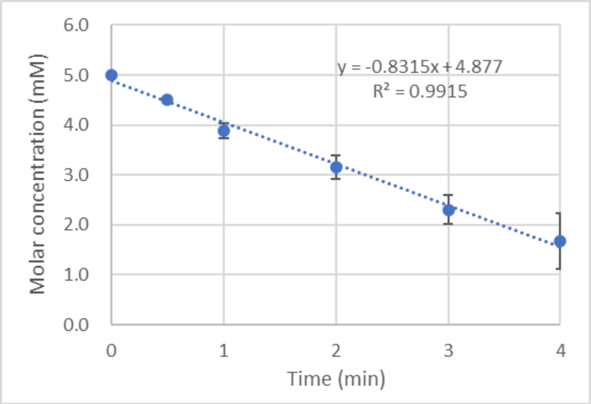

Fig. S5 Time-dependent concentration changes of dacarbazine in the presence of 0.05 equivalents of FAD. Error bars represent standard deviations of triplicates.

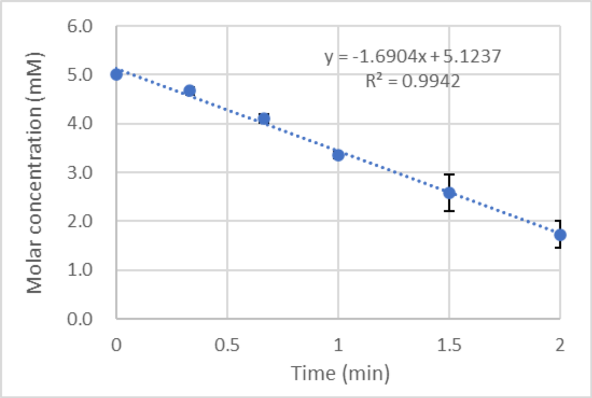

Fig. S6 Time-dependent concentration changes of dacarbazine without riboflavin. Error bars represent standard deviations of triplicates.

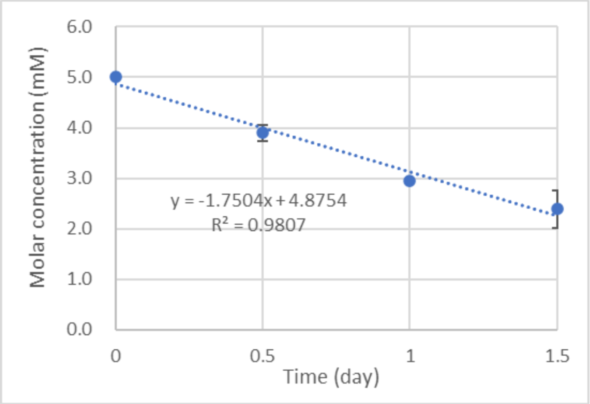

Fig. S7 Time-dependent concentration changes of dacarbazine in the presence of 0.05 equivalents of riboflavin under fluorescent light. Error bars represent standard deviations of triplicates.

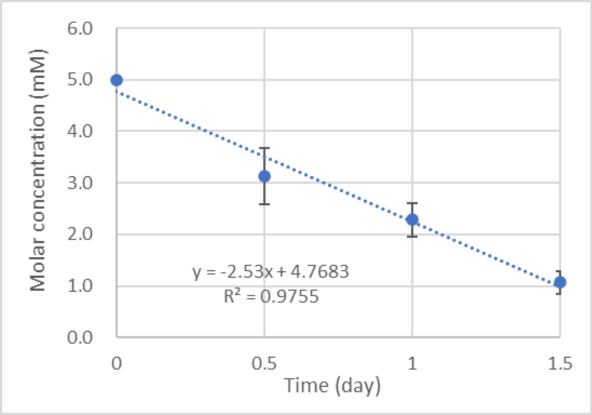

Fig. S8 Time-dependent concentration changes of dacarbazine in the presence of 0.01 equivalents of riboflavin under fluorescent light. Error bars represent standard deviations of triplicates.

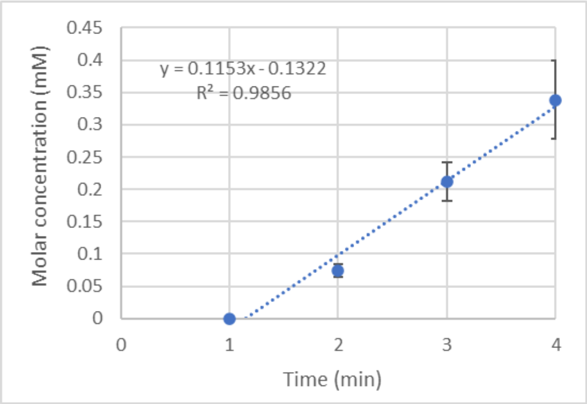

Fig. S9 Time-dependent concentration changes of **2** in the presence of 0.05 equivalents of riboflavin. Error bars represent standard deviations of triplicates.

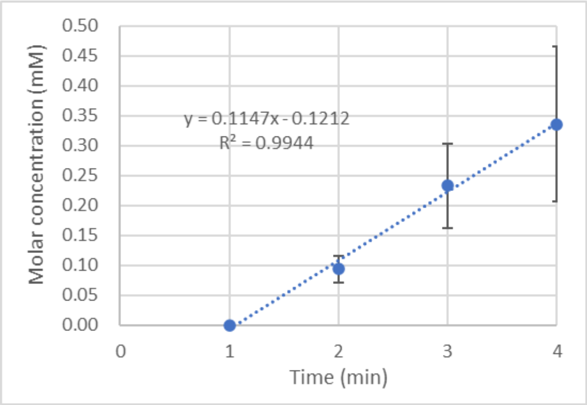

Fig. S10 Time-dependent concentration changes of **2** in the presence of 0.05 equivalents of FAD. Error bars represent standard deviations of triplicates.

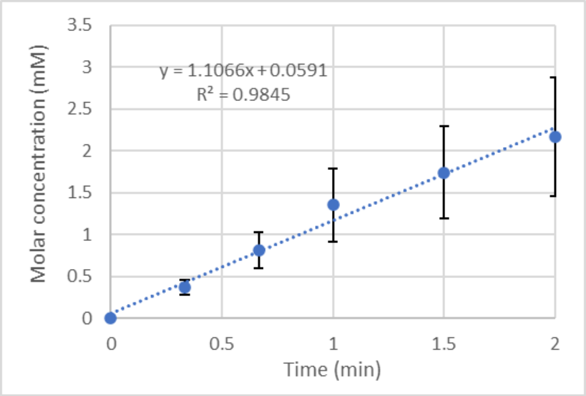

Fig.S11 Time-dependent concentration changes of **2** without riboflavin. Error bars represent standard deviations of triplicates.

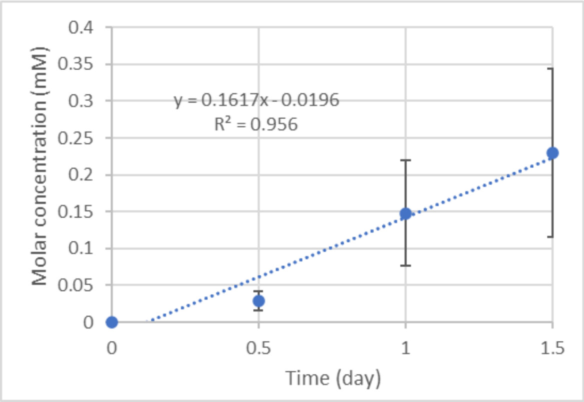

Fig. S12 Time-dependent concentration changes of **2** in the presence of 0.05 equivalents of riboflavin under fluorescent light. Error bars represent standard deviations of triplicates.

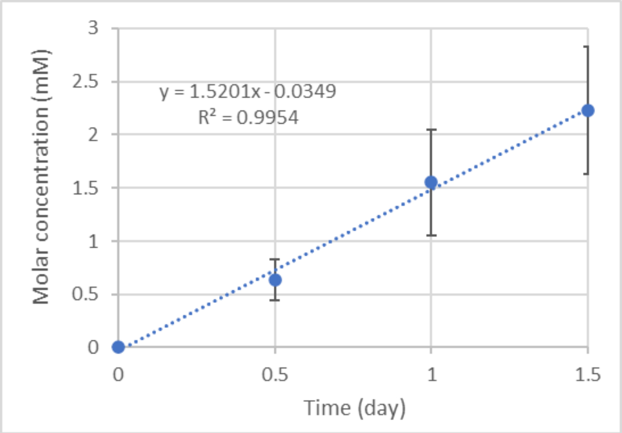

Fig. S13 Time-dependent concentration changes of 2 in the presence of 0.01 equivalents of riboflavin under fluorescent light. Error bars represent standard deviations of triplicates.

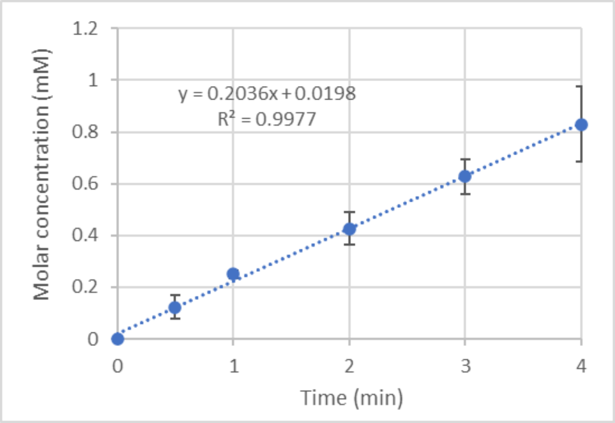

Fig. S14 Time-dependent concentration changes of **6** in the presence of 0.05 equivalents of riboflavin. Error bars represent standard deviations of triplicates.

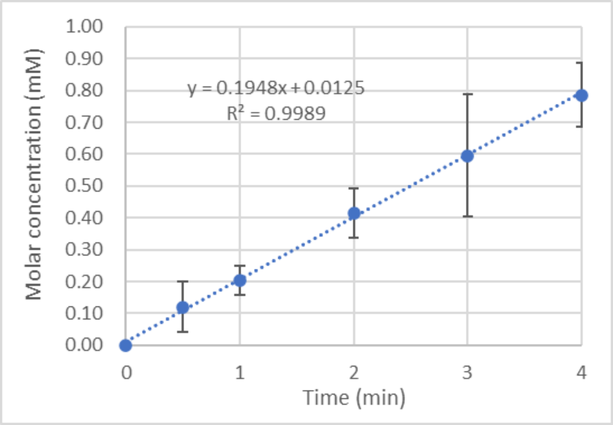

Fig. S15 Time-dependent concentration changes of **6** in the presence of 0.05 equivalents of FAD. Error bars represent standard deviations of triplicates.

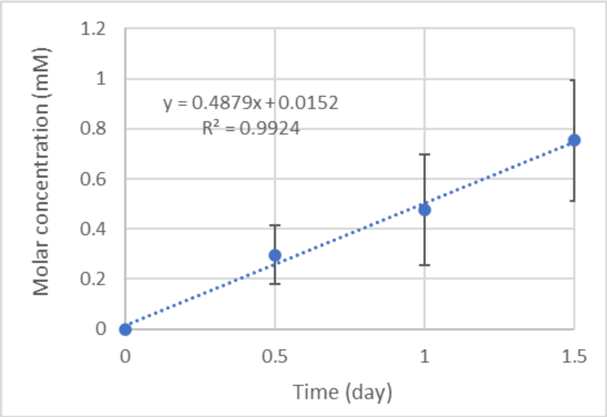

Fig. S16 Time-dependent concentration changes of **6** in the presence of 0.05 equivalents of riboflavin under fluorescent light. Error bars represent standard deviations of triplicates.

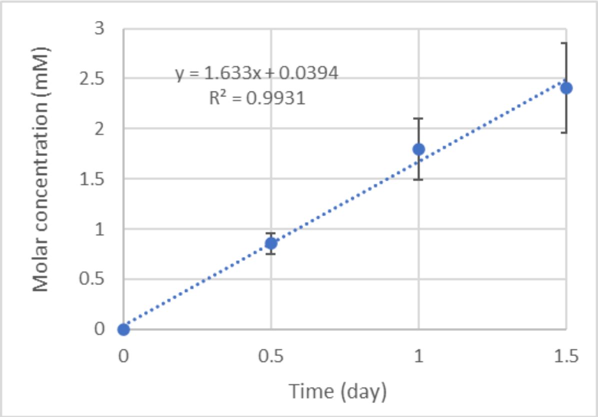

Fig. S17 Time-dependent concentration changes of **6** in the presence of 0.01 equivalents of riboflavin under fluorescent light. Error bars represent standard deviations of triplicates.
